# Deciphering the potential ability of RG108 in cisplatin-induced HEI-OC1 ototoxicity: a research based on RNA-seq and molecular biology experiment

**DOI:** 10.1186/s41065-023-00283-y

**Published:** 2023-04-24

**Authors:** Dongdong Zhang, Yixin Sun, Min Lei, Yue Wang, Chengfu Cai

**Affiliations:** 1 Department of Otolaryngology-Head and Neck Surgery, School of Medicine, Zhongshan Hospital, Xiamen University, Xiamen, 361003 China; 2 School of Medicine, Xiamen University, Xiamen, 361003 China; 3 Department of Surgery, The Second People’s Hospital of Neijiang Affiliated to Southwest Medical University, Neijiang, 641000 China; 4 Department of Surgery, The Second People’s Hospital of Neijiang, Neijiang, 641000 China

**Keywords:** Hearing loss, Ototoxicity, NRF2/PI3K-AKT axis, RG108

## Abstract

**Background:**

Drug-induced hearing loss (DIHL) is very common, and seriously affects people's happiness in life. RG108 is a small molecule inhibitor. RG108 is protective against DIHL. Our purpose is to probe the incidence of RG108 on cisplatin-induced ototoxicity.

**Materials and methods:**

In our research, the ototoxicity of RG108 was investigated in HEI-OC1. We observed under the microscope whether RG108 had an effect on cisplatin-induced cochlear hair cells. RNA-seq experiments were further performed to explore possible gene ontology (GO) and pathways. ROS assay was applied to supervisory the effect of RG108 on oxidative harm of auditory cells. In auditory cells, RG108 was tested for its effects on apoptosis-related proteins by Western blotting (WB).

**Results:**

GO analysis showed that RG108 associated with apoptosis. KEGG analysis shows RG108 may act on PI3K-AKT signaling pathway (PASP) in hearing loss. BIOCARTA analysis showed that RG108 may affect oxidative stress by activating NRF2 pathway. ROS ascerted that RG108 could rescue oxidative harm in HEI-OC1. RG108 rescued cisplatin-induced significant increase in Bax and significant decrease in BCL2. RG108 attenuates cisplatin-induced cochlear apoptosis through upregulated phosphorylated PI3K and phosphorylated AKT and down-regulated caspase3. MTT experiments showed that both PI3K and AKT inhibitors could significantly rescue the damage caused by cisplatin to HEI-OC1. RG108 significantly increases the level of NRF2/HO-1/NQO1 in cisplatin-induced cells.

**Conclusion:**

Overall, these results provide evidence that NRF2/PI3K-AKT axis may mediate RG108 in the treatment of DIHL, which provide a broader outlook on drug-induced deafness treatment.

## Introduction

Globally, hearing loss (HL) is one of the most prevalent diseases, and its incidence is on the rise [1]. The most familiar physiological cause of hearing loss is aging. But there are many other factors associated with hearing loss, such as: medications, trauma, etc. [2]. Cisplatin-induced deafness is usually irreversible, bilateral, progressive, with an incidence of 20% -70% [3–10]. With the increase of tumor incidence, Drug-induced hearing loss (DIHL) population is increasing. DIHL has become the focus and hotspot of ear science research worldwide [11–13]. DIHL is currently a great challenge in the world, and hearing loss significantly affects people’s well-being and emotions, so it has important research significance [14].

Although many studies have proposed the use of different drugs for hearing protection, there are limitations to their effectiveness. In our study, we explored the mechanism of action between RG108 and deafness. We hope that these mechanisms of action will be helpful for the diagnosis and treatment of deafness. RG108 as DNMT1 plays a vital character in many biological behaviour [15]. Previous researches have shown that RG108 reduces oxidative DNA damage and apoptosis of auditory cells after noise exposure, and RG108 has a rescue effect on noise-induced hearing loss [16]. This study mainly focused on the mechanism of action of RG 108 on drug-induced deafness. Apoptosis is considered to be an important part of various processes. However, if normal cells undergo excessive apoptosis, it will affect the normal health of the body [17]. The ototoxicity of drugs is mainly manifested in the promotion of apoptosis of cochlear hair cells [18]. Our study found that RG108 rescues drug-induced cochlear apoptosis to some extent.

A crucial transcription factor, NRF2 regulates cellular oxidative stress responses and plays a crucial role in maintaining cellular redox balance [19, 20]. NFE2L2 encodes the transcription factor Nrf2 [21, 22]. Activation of Nrf2 can mediate antioxidant/anti-inflammatory signaling pathways and affect apoptosis [23]. A number of drugs have been developed to protect against ototoxicity: Curcumin [24], Bucillamine [25], CDDO-Im [26] and so on. PI3K-AKT signaling pathway (PASP) is the predominant apoptosis-related signaling pathway [27]. When the PASP is waked in tumors, it can expedite the occurrence and development of tumors [28–31]. When some specific drugs can act on the PASP, it will affect the apoptosis of cells, thereby affecting the survival of cells. Based on previous achievements, we explored whether RG108 has an effect on the PASP, which in turn affects the apoptosis process of cochlear hair cells.

Genome sequencing technology is currently widely used in scientific research [32, 33], and schools and research institutions around the world are combining their research topics with sequencing. At present, our research group has made full use of next-generation sequencing technology. By analyzing the biological functions and pathways of drugs on cochlear hair cells, we can outline the mechanism of RG108 on deafness at the molecular level.

In this study, we experimentally confirmed that RG108, as a small molecule inhibitor, can rescued cisplatin-induced apoptosis of cochlear hair cells. RNA-seq was used to investigated the mechanism of RG108 inhibiting cisplatin-induced ototoxicity. Further phenotypic experiments, WB and ROS confirmed that RG108 had effect in drug-induced deafness. RG108 regulates NRF2-antioxidative stress signaling, which is ideal for preventing or treating drug-induced deafness.

## Materials and methods

### Acquisition of HEI‐OC1 and RG108

HEI-OC1 (The House Ear Institute-Organ of Corti 1) cells come from mouse, and has been used worldwide to study apoptosis pathway, oxidative stress, inflammation and so on [34]. As a DNA methylation inhibitor, RG108 (Selleck Chemicals, USA; S2821), and it has been used extensively to treat tumors, including esophageal, endometrial, and prostate cancers [35–37], etc. Cisplatin (Selleck Chemicals, USA; S1166), it has been shown that this anti-tumor drug plays a crucial role in solid tumors, such as testicular cancer, breast cancer, [38–41]. HEI-OC1 cells were donated by Fudan University. RG108 and cisplatin were purchased from Selleck Chemicals Biotechnology Co., Ltd.

### Cell culture

HEI-OC1 cell line was cultured in 2.5% fetal bovine serum (Cat: 10099141C) high glucose DMEM (without antibiotics) (SKU: 06–1055-57-1A) (37 °C, 5% CO2). Both RG108 and cisplatin were dissolved in DMSO. As per the instructions of the manufacturer, we conducted a dissolution experiment, and stored at -20 °C using 1.5 ml centrifuge tube. We treated HEI‐OC1 with different titers of DMSO (called control group), cisplatin, cisplatin + RG108, RG108 for 24 h. Observed the state of each group of cells under a light microscope to further explore whether RG108 will affect the phenotype of HEI-OC1. In addition, mycoplasma was detected every month to ensure that the mycoplasma of cultured cells was negative. All cells were cultured in 10 cm culture dish, and the cell density was maintained at 70%, and passage after trypsin (Merck Reagent; CAS: 9002–07-7) digestion.

### RNA-seq

This sequencing was completed by Amor Gene Xiamen Biotechnology Co., Ltd. in Xiamen, Fujian Province, China, and the collection of sequencing samples was completed according to the company's requirements. Each group took 3 replicates. The phenotypic changes of cells in different treatment groups were observed under a microscope (Japan; Olympus). To further explore the mechanism of RG108 on cochlear hair cell, we performed next-generation biological sequencing (mRNA-seq) in different treatment groups. The sequencing experiments were designed as DMSO, cisplatin, RG108, cisplatin + RG108. Three plates of cells were prepared for each tranches. The cells were disposed with DMSO solution (control), 30um cisplatin (CIS), 30um cisplatin and 100um RG108 (CIS + RG108), and 100um RG108. Each group was tested for mycoplasma before collection. The number of cells in each test tube is maintained at 5 × 106 to ensure that no mycoplasma contamination occurs. The cells were collected and placed in test tubes after strict collection procedures, and TRIZOL (Invitrogen; Cat:15596018) was added to store at -80 °C.

### RNA extraction

According to the manufacturer’s requirements, and all RNA was extracted from cells using chloroform reagent. After successful extraction, the RNA was stored in the cryopreserved tube, and the subsequent experiments were carried out by the biological sequencing company.

### Primary data processing

FASTQ (0.19.3) was used for data filtering. STAR (2.7.3a) was used for genome alignment. Annovar (2020–06-07) for genome annotation. RSeQC (v2.6.4) was used for post-comparison quality control. Cufflinks (v2.2.1). Transcript quantification. Statistical analysis/plotting (R 4.0.3) [42].

### Bioinformatics analysis of RNA-seq

Analysis of raw data was done by sequencing companies. Examining the effects of cisplatin treatment group on auditory cells. We first performed the bioinformatic analysis of DMSO group and CIS group, and we set the threshold of differential gene screening as *P* < 0.05, |LOG fold change|≥ 1, fold change was twofold. The obtained differential expressed genes are subjected to the next subsequent analysis. Studying the effect of RG108 treatment on auditory cells. We further performed the bioinformatic analysis of CIS group and CIS + RG108 group, and we set the threshold of differential gene screening as *P* < 0.05, |LOG fold change|≥ 1, fold change was twofold. The obtained differential expressed genes are subjected to the next subsequent analysis. Data is presented in the form of volcano maps and heat maps.

### DAVID database

The DAVID database (https://david.ncifcrf.gov/) is currently widely used in the field of bioinformatics. Due to the complexity and complexity of biological research, the DAIVD database has become the most popular biological function prediction database [43]. GO analysis data Genome enrichment analysis, used to study the gene sets of interest are mainly enriched in those biological processes [44, 45]. KEGG [46] and BIOCARTA analysis are pathway analysis, which is mainly used to study the gene sets of interest are mainly involved in pathways. The selected genes meet *P* < 0.05, |LOG fold change|≥ 2, fold change was twofold.

### STRING database

In order to study the effects of RG108 on auditory cells in more detail. We performed an analysis of cisplatin and cisplatin + 108 group. We further indent the threshold. The selected genes meet *P* < 0.05, |LOG fold change|≥ 3, fold change was twofold. Enter the grouped genes into the STRING database (http://string-db.org) [47] for analysis and set the threshold to the minimum required interaction score: 0.400.

### Cytoscape database and MCODE/Clue-GO plug-in

Cytoscape (https://cytoscape.org/) [48] is a software used to process pictures, which has rich functions. The above TSV file data cytoscape database for subsequent analysis, looking for core genes. MCODE is a plug-in in cytoscape software, which has the algorithm function and can be connected to the public database to analyze the input files. Set the threshold to degree cutoff: 12, K-Core = 4. Clue-GO is a biological function plug-in that comes with cytoscae software, which can perform enrichment analysis on gene sets of interest. We performed enrichment analysis for gene sets that met the following thresholds *P* < 0.05, |LOG fold change|≥ 3, fold change was twofold,The result filter condition is* P* < 0.05.

### Reactive oxygen species (ROS)

Use kits to detect intracellular reactive oxygen species production, using fluorescent probes. Treat cells with suitable concentrations of drugs and dye them with fuel. 2',7'-Dichlorodihydrofluorescein diacetate (DCFH-DA) and Dihydroethidium (DHE) probe functioning solution (10 μ m, Beyotime, S0033) was entried to a six-well plate and the plate was incubated at 37 °C for 30 min. A Leica SP8 laser scanning confocal microscope was used to observe fluorescence, and Image J was used to quantify strong and weak fluorescence.

### Western blot

The treated cells were collected and protein was extracted for quantitative analysis.

The adherent cells were washed three times with PBS, digested with trypsin, centrifuged, and collected into the centrifuge tube. According to the number of cells to join the appropriate amount of RIPA ( mixed with PMSF and cocktail) (Cloudy sky; cat: P0013C), ice bath 30 min, 12000 g centrifugal 5 min, collect supernatant for protein. Using the BCA protein concentration determination kit, the protein concentration was determined. The HEI-OC1 was separated by 10% SDS polyacrylamide gel and transferred to polyvinylidene fluoride membranes. Further blocked with 5% BSA, and incubated with anti *GAPDH, ACTIN, NRF2, BCL2, BAX, P-PI3K, P-AKT, PI3K,Caspase3,HO-1,NQO1 a*nd *AKT* (Cell Signaling Technology) for 4 degrees overnight (All antibody configurations follow manufacturer instructions). All antibodies were purchased from sigma Aldrich and were responsible by Xiamen agents in China. Oxidase coupled secondary antibody (sigma Aldrich, 1:1000 dilution, 2 h, room temperature shaking table) was added, and the signal was obtained using ECL system (sigma Aldrich). ImageJ software was used to quantify protein bands. GAPDH and actin were used as loading controls to standardize relative expression.

### Statistical analysis

*P*-values < 0.05 was statistically significant. In order to show the difference between the two groups by statistical significance, we use the statistical method used was ANOVA or unpaired student's t test. The calculation software used is Graphpad Prism 8. Analyses were conducted with R software (version 3.6.3) [49].

## Results

### RG108 pretreatment significantly reduced the damage of HEI-OC1 induced by cisplatin

**Fig. 1:**
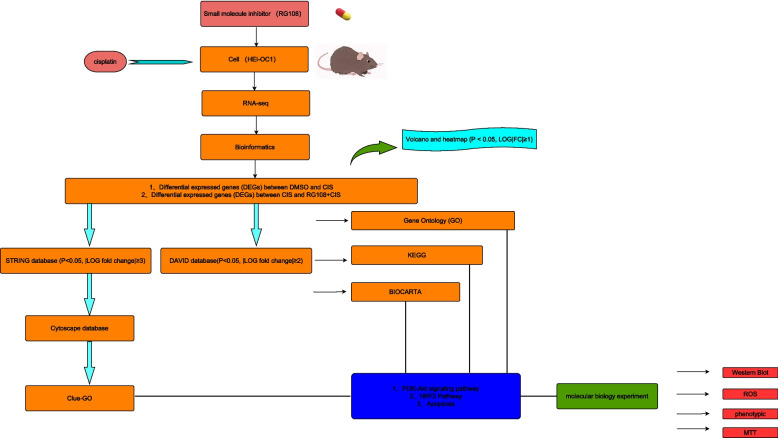
The schematic flow chart of the study

**Fig. 2:**
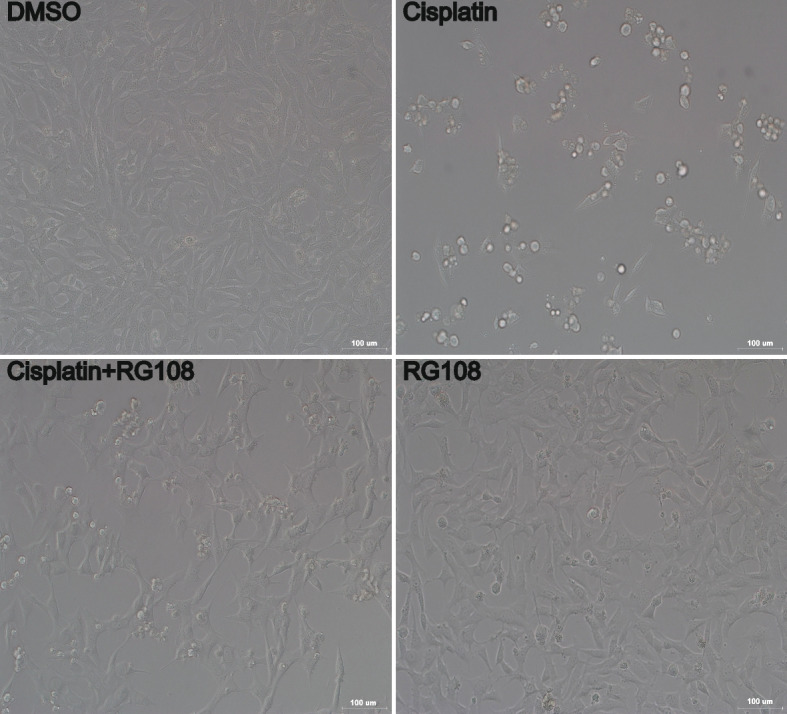
Drug-induced changes in cell state. The morphological changes of HEI-OC1 under different treatment conditions were observed in the open field. scale: 100 μm

The research steps of this manuscript are presented in the form of a flow chart in Fig. 1. We use ordinary microscope to observe the morphological changes of HEI-OC1 in different treatment groups. The results are obvious. The morphology of HEI-OC1 cells in cisplatin injury group has changed significantly compared with the control group. Of course, there is no obvious morphological abnormality in RG108 pretreatment group compared with cisplatin injury group. We can see from the pictures of the bright field that the cell morphology of the RG108 pretreated group is significantly better than that of the cisplatin group (Fig. 2).

### RNA-seq analysis

**Fig. 3:**
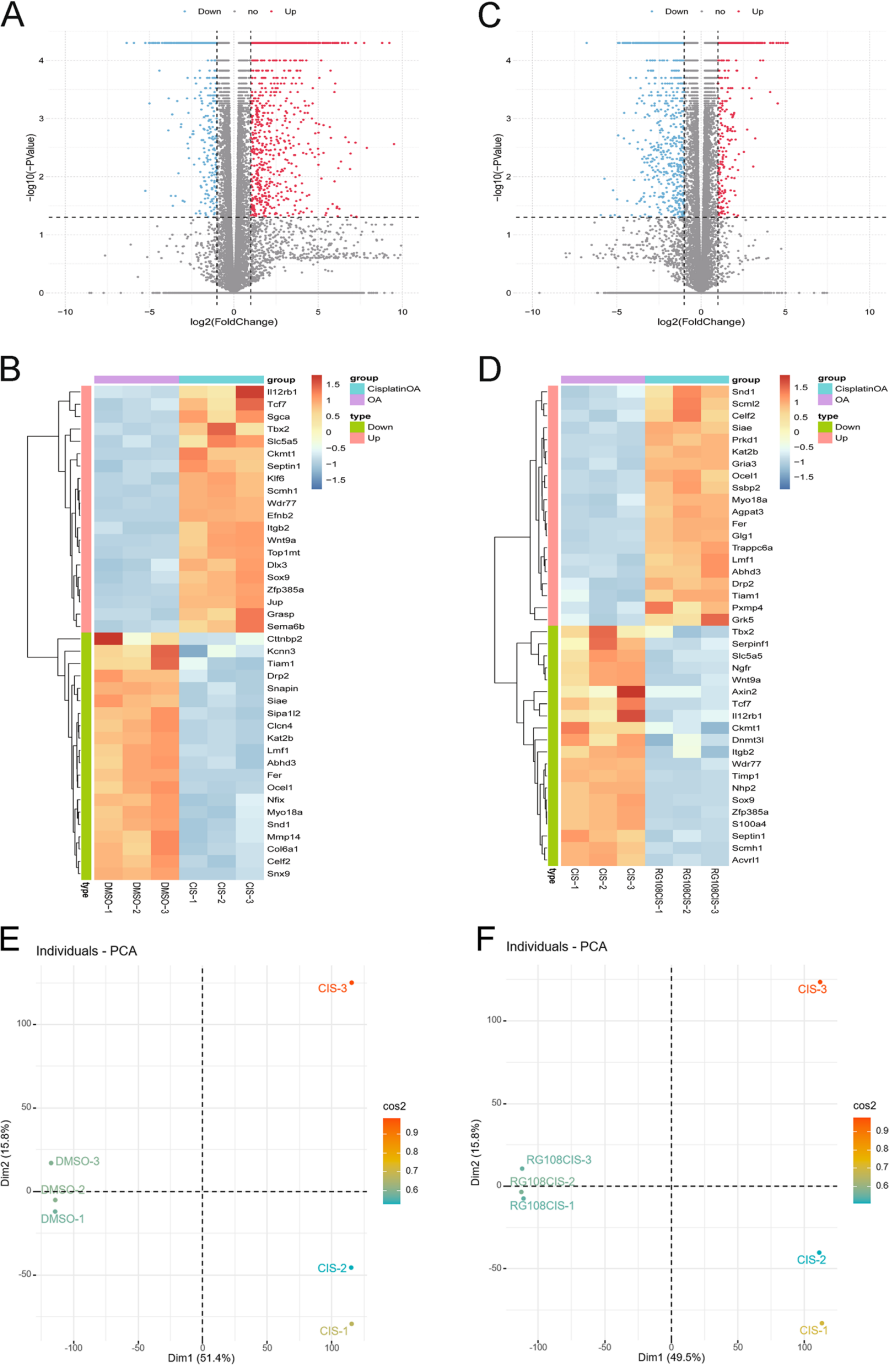
Volcano plot and heatmap of DEGs. **A** Volcano plot of DEGs. DEGs between DMSO and CIS. **B** Heatmap of DEGs. DEGs between DMSO and CIS. **C** Volcano plot of DEGs. DEGs between CIS and CIS + RG108. **D** Heatmap of DEGs. DEGs between CIS and CIS + RG108. **E** PCA analysis result of DMSO and CIS. **F** PCA analysis result of CIS and CIS + RG108

Analysis of raw data was done by sequencing companies. Comparing the control group with the CIS, we obtained a total of 2539 differentially expressed genes (DEGs), of which 1525 genes were up-regulated and 1014 genes were down-regulated. Comparing CIS with CIS + RG108, we obtained a total of 583 DEGs, of which 173 genes were up-regulated and 410 genes were down-regulated. Threshold was set at *P* < 0.05, |LOG2 fold change|≥ 1. DEGs were displayed in the form of volcano plots and heat maps, respectively (Fig. 3A-D). Principal component analysis (PCA) of each sequencing sample is shown in Fig. 3E-F.

### GO, KEGG and BIOCARTA analysis of DMSO group and CIS group

**Fig. 4:**
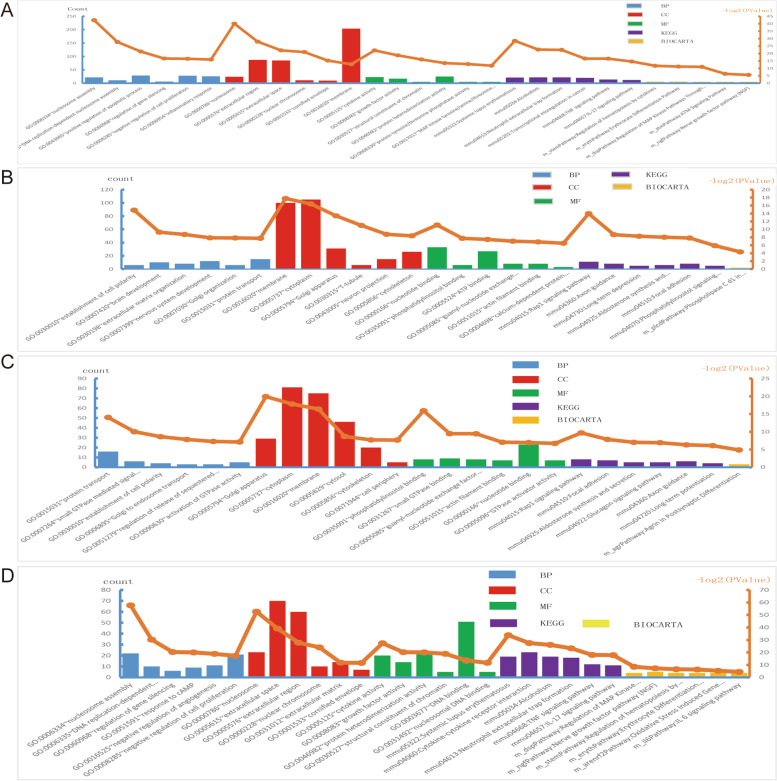
Gene ontology (GO), Kyoto Encyclopedia of Genes and Genomes (KEGG) and BIOCARTA analysis of DEGs. BP: Biological Process, CC: cellular component, MF: molecular function, KEGG: Kyoto Encyclopedia of Genes and Genomes. **A** GO, KEGG and BIOCARTA analysis between DMSO and CIS in UP-DEGs.) GO, KEGG and BIOCARTA analysis between DMSO and CIS in Down-DEGs. **C** GO, KEGG and BIOCARTA analysis between CIS and CIS + RG108 in UP-DEGs. **D** GO, KEGG and BIOCARTA analysis between CIS and CIS + RG108 in Down-DEGs

We used the DAVID database to analyze DEGs, and threshold was set at *P* < 0.05, |LOG2 fold change|≥ 2. Compared DMSO group with cisplatin group, the up-DEGs were enriched in nucleosome assembly, DNA replication-dependent nucleosome assembly, positive regulation of apoptotic process, regulation of gene silencing, negative regulation of cell proliferation, and inflammatory response in BP. Up-DEGs were enriched in nucleosome, extracellular region, extracellular space, nuclear chromosome, cornified envelope, and membrane in CC. Up-DEGs were enriched in cytokine activity, growth factor activity, structural constituent of chromatin, protein heterodimerization activity, protein tyrosine/threonine phosphatase activity, and MAP kinase tyrosine/serine/threonine phosphatase activity in MF. Up-DEGs were enriched in Systemic lupus erythematosus, Alcoholism, Neutrophil extracellular trap formation, Transcriptional misregulation in cancer, TNF signaling pathway, and IL-17 signaling pathway in KEGG. Up-DEGs were enriched in Regulation of hematopoiesis by cytokines, Erythrocyte Differentiation Pathway, Regulation of MAP Kinase Pathways Through Dual Specificity Phosphatases, ATM Signaling Pathway, and Nerve growth factor pathway (NGF) in BIOCARTA (Table 1) (Fig. 4A). Compared DMSO group with cisplatin group, the down-DEGs were enriched in establishment of cell polarity, brain development, extracellular matrix organization, nervous system development, Golgi organization, and protein transport in BP. Down-DEGs were enriched in membrane, cytoplasm, Golgi apparatus, T-tubule, neuron projection, and cytoskeleton in CC. Down-DEGs were enriched in nucleotide binding, phosphatidylinositol binding, ATP binding, guanyl-nucleotide exchange factor activity, actin filament binding, and calcium-dependent protein kinase C activity in MF. Down-DEGs were enriched in Rap1 signaling pathway, Axon guidance, Long-term depression, Aldosterone synthesis and secretion, Focal adhesion, and Phosphatidylinositol signaling system in KEGG. Down-DEGs were enriched in Phospholipase C d1 in phospholipid associated cell signaling in BIOCARTA (Table2) (Fig. 4B).

**Table 1:** GO, KEGG and BIOCARTA analysis based on DAVID. (Up-DEGs between DMSO with CIS)

GO	Category	Count	-Log2(*P* Value)	
GOTERM_BP_DIRECT	GO:0006334 ~ nucleosome assembly	22	42.403049	
GOTERM_BP_DIRECT	GO:0006335 ~ DNA replication-dependent nucleosome assembly	11	27.74878128	
GOTERM_BP_DIRECT	GO:0043065 ~ positive regulation of apoptotic process	29	21.32817221	
GOTERM_BP_DIRECT	GO:0060968 ~ regulation of gene silencing	6	16.60892704	
GOTERM_BP_DIRECT	GO:0008285 ~ negative regulation of cell proliferation	28	16.45457063	
GOTERM_BP_DIRECT	GO:0006954 ~ inflammatory response	26	15.93301132	
GOTERM_BP_DIRECT	GO:0000786 ~ nucleosome	24	40.02493217	
GOTERM_BP_DIRECT	GO:0005576 ~ extracellular region	87		27.93206549
GOTERM_BP_DIRECT	GO:0005615 ~ extracellular space	85	22.10672588	
GOTERM_BP_DIRECT	GO:0000228 ~ nuclear chromosome	11	20.94169347	
GOTERM_CC_DIRECT	GO:0001533 ~ cornified envelope	10	15.2297727	
GOTERM_CC_DIRECT	GO:0016020 ~ membrane	204		12.79847186
GOTERM_CC_DIRECT	GO:0005125 ~ cytokine activity	23	22.05968309	
GOTERM_CC_DIRECT	GO:0008083 ~ growth factor activity	17	18.742172	
GOTERM_CC_DIRECT	GO:0030527 ~ structural constituent of chromatin	5	15.99923777	
GOTERM_CC_DIRECT	GO:0,046,982 ~ protein heterodimerization activity	25	13.51717047	
GOTERM_CC_DIRECT	GO:0008330 ~ protein tyrosine/threonine phosphatase activity	5	12.88292008	
GOTERM_CC_DIRECT	GO:0017017 ~ MAP kinase tyrosine/serine/threonine phosphatase activity	5	11.8276452	
GOTERM_CC_DIRECT	mmu05322:Systemic lupus erythematosus	21	28.48228567	
GOTERM_CC_DIRECT	mmu05034:Alcoholism	22	22.65126955	
GOTERM_MF_DIRECT	mmu04613:Neutrophil extracellular trap formation	22	22.41079607	
GOTERM_MF_DIRECT	mmu05202:Transcriptional misregulation in cancer	20	16.57777767	
GOTERM_MF_DIRECT	mmu04668:TNF signaling pathway	14	16.49493421	
GOTERM_MF_DIRECT	mmu04657:IL-17 signaling pathway	12	14.60610374	
GOTERM_MF_DIRECT	m_stemPathway:Regulation of hematopoiesis by cytokines	6	11.73475312	
GOTERM_MF_DIRECT	m_erythPathway:Erythrocyte Differentiation Pathway	6	11.2031028	
GOTERM_MF_DIRECT	m_dspPathway:Regulation of MAP Kinase Pathways Through Dual Specificity Phosphatases	5	11.01320069	
GOTERM_MF_DIRECT	m_atmPathway:ATM Signaling Pathway	5	6.317244644	
GOTERM_MF_DIRECT	m_ngfPathway:Nerve growth factor pathway (NGF)	5	5.6012101	
GOTERM_MF_DIRECT	GO:0006334 ~ nucleosome assembly	22	42.403049	
KEGG_PATHWAY	GO:0006335 ~ DNA replication-dependent nucleosome assembly	11	27.74878128	
KEGG_PATHWAY	GO:0043065 ~ positive regulation of apoptotic process	29	21.32817221	
KEGG_PATHWAY	GO:0060968 ~ regulation of gene silencing	6	16.60892704	
KEGG_PATHWAY	GO:0008285 ~ negative regulation of cell proliferation	28	16.45457063	
KEGG_PATHWAY	GO:0006954 ~ inflammatory response	26	15.93301132	
KEGG_PATHWAY	GO:0000786 ~ nucleosome	24	40.02493217	
KEGG_PATHWAY	GO:0005576 ~ extracellular region	87	27.93206549	
KEGG_PATHWAY	GO:0005615 ~ extracellular space	85	22.10672588	
KEGG_PATHWAY	GO:0000228 ~ nuclear chromosome	11	20.94169347	
KEGG_PATHWAY	GO:0001533 ~ cornified envelope	10	15.2297727	
BIOCARTA	GO:0016020 ~ membrane	204	12.79847186	
BIOCARTA	GO:0005125 ~ cytokine activity	23	22.05968309	
BIOCARTA	GO:0008083 ~ growth factor activity	17	18.742172	
BIOCARTA	GO:0030527 ~ structural constituent of chromatin	5	15.99923777	
BIOCARTA	GO:0046982 ~ protein heterodimerization activity	25	13.51717047	
BIOCARTA	GO:0008330 ~ protein tyrosine/threonine phosphatase activity	5	12.88292008	
BIOCARTA	GO:0017017 ~ MAP kinase tyrosine/serine/threonine phosphatase activity	5	11.8276452

**Table 2:** GO, KEGG and BIOCARTA analysis based on DAVID. (Down-DEGs between DMSO with CIS)

GO	Category	Count	-Log2(*P* Value)
GOTERM_BP_DIRECT	GO:0030010 ~ establishment of cell polarity	6	4.84265555
GOTERM_BP_DIRECT	GO:0007420 ~ brain development	10	9.291692541
GOTERM_BP_DIRECT	GO:0030198 ~ extracellular matrix organization	8	8.723977207
GOTERM_BP_DIRECT	GO:0007399 ~ nervous system development	12	7.879202652
GOTERM_BP_DIRECT	GO:0007030 ~ Golgi organization	6	7.848307704
GOTERM_BP_DIRECT	GO:0015031 ~ protein transport	15	7.756581427
GOTERM_BP_DIRECT	GO:0016020 ~ membrane	100	17.7794164
GOTERM_BP_DIRECT	GO:0005737 ~ cytoplasm	105	16.41697626
GOTERM_BP_DIRECT	GO:0005794 ~ Golgi apparatus	31	13.40458113
GOTERM_BP_DIRECT	GO:0030315 ~ T-tubule	6	11.00613272
GOTERM_CC_DIRECT	GO:0043005 ~ neuron projection	15	8.74749388
GOTERM_CC_DIRECT	GO:0005856 ~ cytoskeleton	26	8.386444372
GOTERM_CC_DIRECT	GO:0000166 ~ nucleotide binding	33	11.0905486
GOTERM_CC_DIRECT	GO:0035091 ~ phosphatidylinositol binding	6	7.733881177
GOTERM_CC_DIRECT	GO:0005524 ~ ATP binding	27	7.467695552
GOTERM_CC_DIRECT	GO:0005085 ~ guanyl-nucleotide exchange factor activity	8	7.030604737
GOTERM_CC_DIRECT	GO:0051015 ~ actin filament binding	8	6.842845867
GOTERM_CC_DIRECT	GO:0004698 ~ calcium-dependent protein kinase C activity	3	6.508653954
GOTERM_CC_DIRECT	mmu04015:Rap1 signaling pathway	11	13.97604962
GOTERM_CC_DIRECT	mmu04360:Axon guidance	8	8.657491879
GOTERM_MF_DIRECT	mmu04730:Long-term depression	5	8.273286919
GOTERM_MF_DIRECT	mmu04925:Aldosterone synthesis and secretion	6	8.025647526
GOTERM_MF_DIRECT	mmu04510:Focal adhesion	8	7.819253967
GOTERM_MF_DIRECT	mmu04070:Phosphatidylinositol signaling system	5	5.905605906
GOTERM_MF_DIRECT	m_plcdPathway:Phospholipase C d1 in phospholipid associated cell signaling	2	4.333272899
GOTERM_MF_DIRECT	GO:0030010 ~ establishment of cell polarity	6	14.84265555
GOTERM_MF_DIRECT	GO:0007420 ~ brain development	10	9.291692541
GOTERM_MF_DIRECT	GO:0030198 ~ extracellular matrix organization	8	8.723977207
GOTERM_MF_DIRECT	GO:0007399 ~ nervous system development	12	7.879202652
GOTERM_MF_DIRECT	GO:0007030 ~ Golgi organization	6	7.848307704
KEGG_PATHWAY	GO:0015031 ~ protein transport	15	7.756581427
KEGG_PATHWAY	GO:0016020 ~ membrane	100	17.7794164
KEGG_PATHWAY	GO:0005737 ~ cytoplasm	105	16.41697626
KEGG_PATHWAY	GO:0005794 ~ Golgi apparatus	31	13.40458113
KEGG_PATHWAY	GO:0030315 ~ T-tubule	6	11.00613272
KEGG_PATHWAY	GO:0043005 ~ neuron projection	15	8.74749388
KEGG_PATHWAY	GO:0005856 ~ cytoskeleton	26	8.386444372
KEGG_PATHWAY	GO:0000166 ~ nucleotide binding	33	11.0905486
KEGG_PATHWAY	GO:0035091 ~ phosphatidylinositol binding	6	7.733881177
KEGG_PATHWAY	GO:0005524 ~ ATP binding	27	7.467695552
BIOCARTA	GO:0005085 ~ guanyl-nucleotide exchange factor activity	8	7.030604737

### GO, KEGG and BIOCARTA analysis of CIS group and CIS + RG108 group

Compared CIS group with CIS + RG108 group, the up-DEGs were mainly fasten in protein transport, small GTPase mediated signal transduction, establishment of cell polarity, Golgi to endosome transport, regulation of release of sequestered calcium ion into cytosol, and activation of GTPase activity in BP. Up-DEGs were mainly fasten in Golgi apparatus, cytoplasm, membrane, cytosol, cytoskeleton, and cell periphery in CC. Up-DEGs were mainly fasten in phosphatidylinositol binding, small GTPase binding, guanyl-nucleotide exchange factor activity, actin filament binding, nucleotide binding, and GTPase activator activity in MF. Up-DEGs were mainly fasten in Rap1 signaling pathway, Focal adhesion, Aldosterone synthesis and secretion, Glucagon signaling pathway, Axon guidance, and Long-term potentiation in KEGG. Up-DEGs were mainly fasten in Agrin in Postsynaptic Differentiation in BIOCARTA (Table 3) (Fig. 4C). Compared CIS group with CIS + RG108 group, the down-DEGs were mainly fasten in nucleosome assembly, DNA replication-dependent nucleosome assembly, regulation of gene silencing, response to cAMP, negative regulation of angiogenesis, and negative regulation of cell proliferation in BP. Down-DEGs were mainly fasten in nucleosome, extracellular space, extracellular region, nuclear chromosome, extracellular matrix, and cornified envelope in CC. Down-DEGs were mainly fasten in cytokine activity, growth factor activity, protein heterodimerization activity, structural constituent of chromatin, DNA binding, and nucleosomal DNA binding in MF. Down-DEGs were mainly fasten in mmu05322:Systemic lupus erythematosus, Cytokine-cytokine receptor interaction, Alcoholism, Neutrophil extracellular trap formation, TNF signaling pathway, IL-17 signaling pathway, Viral carcinogenesis, Rheumatoid arthritis, Transcriptional misregulation in cancer, MAPK signaling pathway, cAMP signaling pathway, Estrogen signaling pathway, PI3K-Akt signaling pathway, Fluid shear stress and atherosclerosis, Staphylococcus aureus infection, Viral protein interaction with cytokine and cytokine receptor, AGE-RAGE signaling pathway in diabetic complications, and Amphetamine addiction in KEGG. Down-DEGs were mainly fasten in Regulation of MAP Kinase Pathways Through Dual Specificity Phosphatases, Nerve growth factor pathway (NGF), Regulation of hematopoiesis by cytokines, Erythrocyte Differentiation Pathway, Oxidative Stress Induced Gene Expression Via NRF2, and IL 6 signaling pathway in BIOCARTA (Table 4) (Fig. 4D). We are more concerned about the PI3K-AKT and NRF2 signaling pathways, because these two pathways have been confirmed to be closely related to apoptosis, and the occurrence of deafness is auditory cell apoptosis. Based on the above findings, we wanted to further confirm whether RG108 could rescue cisplatin-induced apoptosis.

**Table 3:** GO, KEGG and BIOCARTA analysis based on DAVID. (Up-DEGs between CIS with CIS + RG108)

GO	Category	Count	-Log2(*P* Value)
GOTERM_BP_DIRECT	GO:0015031 ~ protein transport	16	14.06367655
GOTERM_BP_DIRECT	GO:0007264 ~ small GTPase mediated signal transduction 6	10.02721346	
GOTERM_BP_DIRECT	GO:0030010 ~ establishment of cell polarity	4	8.62636484
GOTERM_BP_DIRECT	GO:0006895 ~ Golgi to endosome transport	3	7.832803239
GOTERM_BP_DIRECT	GO:0051279 ~ regulation of release of sequestered calcium ion into cytosol	3	7.273426947
GOTERM_BP_DIRECT	GO:0090630 ~ activation of GTPase activity	5	7.128440831
GOTERM_BP_DIRECT	GO:0005794 ~ Golgi apparatus	29	19.91688185
GOTERM_BP_DIRECT	GO:0005737 ~ cytoplasm	81	17.832984
GOTERM_BP_DIRECT	GO:0016020 ~ membrane	75	16.411267
GOTERM_BP_DIRECT	GO:0005829 ~ cytosol	46	8.691662953
GOTERM_CC_DIRECT	GO:0005856 ~ cytoskeleton	20	7.708253097
GOTERM_CC_DIRECT	GO:0071944 ~ cell periphery	5	7.673792745
GOTERM_CC_DIRECT	GO:0035091 ~ phosphatidylinositol binding	8	15.93080634
GOTERM_CC_DIRECT	GO:0031267 ~ small GTPase binding	9	9.466179022
GOTERM_CC_DIRECT	GO:0005085 ~ guanyl-nucleotide exchange factor activity	89.440037152	
GOTERM_CC_DIRECT	GO:0051015 ~ actin filament binding	7	7.061705217
GOTERM_CC_DIRECT	GO:0000166 ~ nucleotide binding	23	6.964765007
GOTERM_CC_DIRECT	GO:0005096 ~ GTPase activator activity	7	6.72004668
GOTERM_CC_DIRECT	mmu04015:Rap1 signaling pathway	8	9.690665567
GOTERM_CC_DIRECT	mmu04510:Focal adhesion	7	7.858315603
GOTERM_MF_DIRECT	mmu04925:Aldosterone synthesis and secretion	5	7.025230162
GOTERM_MF_DIRECT	mmu04922:Glucagon signaling pathway	5	6.927963163
GOTERM_MF_DIRECT	mmu04360:Axon guidance	6	6.328858772
GOTERM_MF_DIRECT	mmu04720:Long-term potentiation	4	6.097971446
GOTERM_MF_DIRECT	m_agrPathway:Agrin in Postsynaptic Differentiation	3	4.881046309
GOTERM_MF_DIRECT	GO:0015031 ~ protein transport	16	14.06367655
GOTERM_MF_DIRECT	GO:0007264 ~ small GTPase mediated signal transduction	6	10.02721346
GOTERM_MF_DIRECT	GO:0030010 ~ establishment of cell polarity	4	8.62636484
GOTERM_MF_DIRECT	GO:0006895 ~ Golgi to endosome transport	3	7.832803239
GOTERM_MF_DIRECT	GO:0051279 ~ regulation of release of sequestered calcium ion into cytosol	3	7.273426947
KEGG_PATHWAY	GO:0090630 ~ activation of GTPase activity	5	7.128440831
KEGG_PATHWAY	GO:0005794 ~ Golgi apparatus	29	19.91688185
KEGG_PATHWAY	GO:0005737 ~ cytoplasm	81	17.832984
KEGG_PATHWAY	GO:0016020 ~ membrane	75	16.411267
KEGG_PATHWAY	GO:0005829 ~ cytosol	46	8.691662953
KEGG_PATHWAY	GO:0005856 ~ cytoskeleton	20	7.708253097
KEGG_PATHWAY	GO:0071944 ~ cell periphery	5	7.673792745
KEGG_PATHWAY	GO:0035091 ~ phosphatidylinositol binding	8	15.93080634
KEGG_PATHWAY	GO:0031267 ~ small GTPase binding	9	9.466179022
KEGG_PATHWAY	GO:0005085 ~ guanyl-nucleotide exchange factor activity	8	9.440037152
BIOCARTA	GO:0051015 ~ actin filament binding	7	7.061705217

**Table 4:** GO, KEGG and BIOCARTA analysis based on DAVID. (Down-DEGs between CIS with CIS + RG108)

GO	Category	Count	-Log2(*P* Value)
GOTERM_BP_DIRECT	GO:0006334 ~ nucleosome assembly	22	57.66454231
GOTERM_BP_DIRECT	GO:0006335 ~ DNA replication-dependent nucleosome assembly	10	30.27275398
GOTERM_BP_DIRECT	GO:0060968 ~ regulation of gene silencing	6	20.33188326
GOTERM_BP_DIRECT	GO:0051591 ~ response to cAMP	9	19.9964746
GOTERM_BP_DIRECT	GO:0016525 ~ negative regulation of angiogenesis	11	18.86607398
GOTERM_BP_DIRECT	GO:0008285 ~ negative regulation of cell proliferation	21	17.30607279
GOTERM_BP_DIRECT	GO:0000786 ~ nucleosome	23	52.68081271
GOTERM_BP_DIRECT	GO:0005615 ~ extracellular space	70	39.16518262
GOTERM_BP_DIRECT	GO:0005576 ~ extracellular region	60	27.78074552
GOTERM_BP_DIRECT	GO:0000228 ~ nuclear chromosome	10	24.06682693
GOTERM_CC_DIRECT	GO:0031012 ~ extracellular matrix	14	11.84546899
GOTERM_CC_DIRECT	GO:0001533 ~ cornified envelope 7 11.55713079		
GOTERM_CC_DIRECT	GO:0005125 ~ cytokine activity	20	27.37639108
GOTERM_CC_DIRECT	GO:0008083 ~ growth factor activity	14	20.21950581
GOTERM_CC_DIRECT	GO:0046982 ~ protein heterodimerization activity	22	20.09644873
GOTERM_CC_DIRECT	GO:0030527 ~ structural constituent of chromatin	5	18.90260936
GOTERM_CC_DIRECT	GO:0003677 ~ DNA binding	51	13.59133025
GOTERM_CC_DIRECT	GO:0031492 ~ nucleosomal DNA binding	5	11.73647731
GOTERM_CC_DIRECT	mmu05322:Systemic lupus erythematosus	19	33.78373863
GOTERM_CC_DIRECT	mmu04060:Cytokine-cytokine receptor interaction	23	27.47096385
GOTERM_MF_DIRECT	mmu05034:Alcoholism	19	26.09629208
GOTERM_MF_DIRECT	mmu04613:Neutrophil extracellular trap formation	18	23.29859253
GOTERM_MF_DIRECT	mmu04668:TNF signaling pathway	12	17.9973151
GOTERM_MF_DIRECT	mmu04657:IL-17 signaling pathway	11	17.81081119
GOTERM_MF_DIRECT	m_dspPathway:Regulation of MAP Kinase Pathways Through Dual Specificity Phosphatases	4	8.521766816
GOTERM_MF_DIRECT	m_ngfPathway:Nerve growth factor pathway (NGF)	5	7.250046074
GOTERM_MF_DIRECT	m_stemPathway:Regulation of hematopoiesis by cytokines	4	6.579771454
GOTERM_MF_DIRECT	m_erythPathway:Erythrocyte Differentiation Pathway	4	6.292419207
GOTERM_MF_DIRECT	m_arenrf2Pathway:Oxidative Stress Induced Gene Expression Via Nrf2	4	5.339674577
GOTERM_MF_DIRECT	m_il6Pathway:IL 6 signaling pathway	4	4.448413649
KEGG_PATHWAY	GO:0006334 ~ nucleosome assembly	22	57.66454231
KEGG_PATHWAY	GO:0006335 ~ DNA replication-dependent nucleosome assembly	10	30.27275398
KEGG_PATHWAY	GO:0060968 ~ regulation of gene silencing	6	20.33188326
KEGG_PATHWAY	GO:0051591 ~ response to cAMP	9	19.9964746
KEGG_PATHWAY	GO:0016525 ~ negative regulation of angiogenesis	11	18.86607398
KEGG_PATHWAY	GO:0008285 ~ negative regulation of cell proliferation	21	17.30607279
KEGG_PATHWAY	GO:0000786 ~ nucleosome	23	52.68081271
KEGG_PATHWAY	GO:0005615 ~ extracellular space	70	39.16518262
KEGG_PATHWAY	GO:0005576 ~ extracellular region	60	27.78074552
KEGG_PATHWAY	GO:0000228 ~ nuclear chromosome	10	24.06682693
BIOCARTA	GO:0031012 ~ extracellular matrix	14	11.84546899

### STRING analysis

**Fig. 5:**
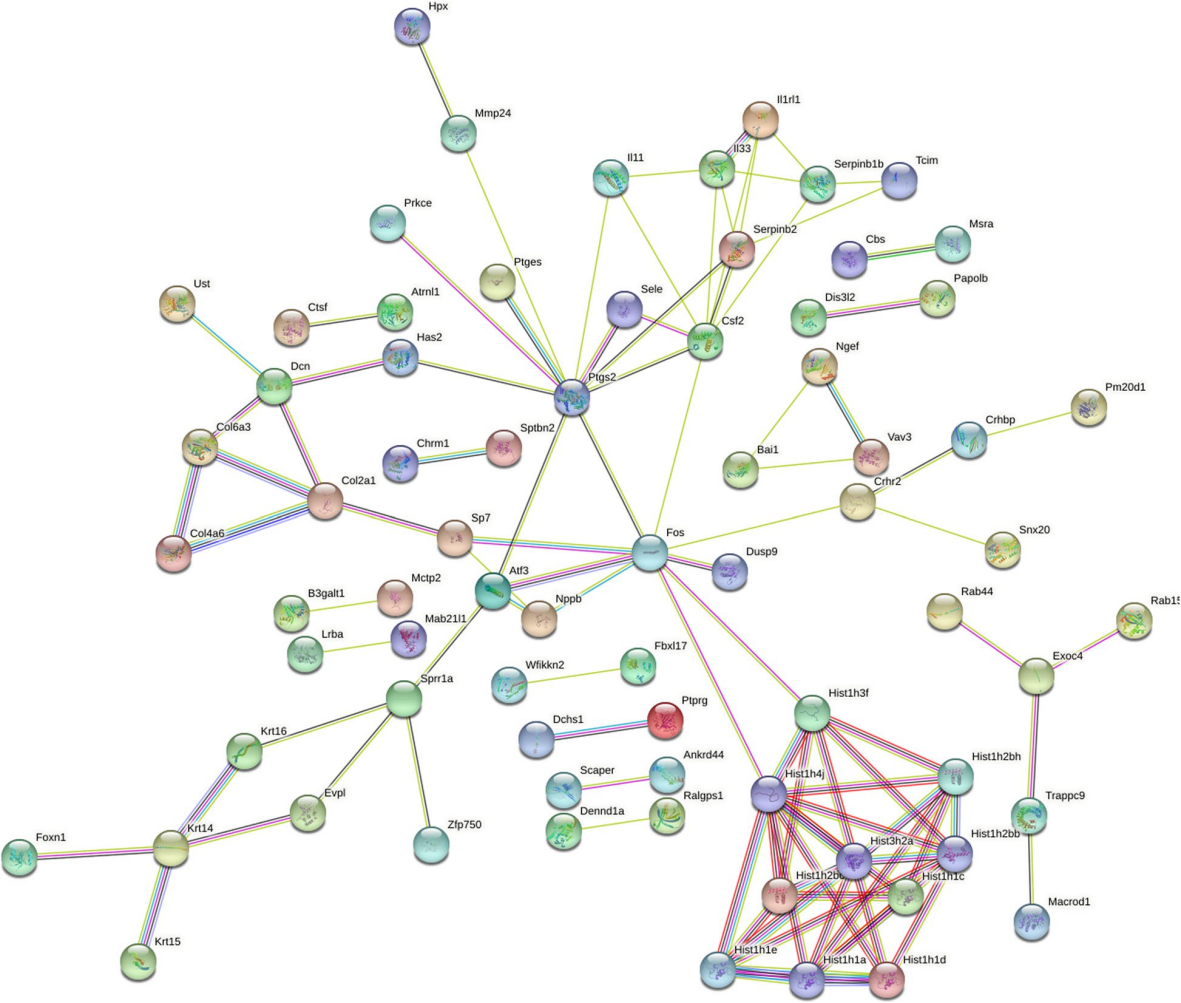
Protein–protein interaction network analysis in STRING database

To explore the pivotal genes that RG108 plays a protective role in drug-induced deafness, we imported 184 genes into the STRING online database, including 44 up-regulated genes and 140 down-regulated genes (Table 5). Protein–protein interaction detection was performed on these genes, and some genes with low correlation were filtered out. The results are shown in Fig. 5. The output is number of nodes: 152, number of edges:102, average node degree:1.34, avg. local clustering coefficient: 0.337, expected number of edges:53, and PPI enrichment *p*-value: 1.49e-09.

**Table 5:** One hundred eighty-four Differentially Expressed Genes (DEGs) were identified from CIS vs CIS + RG108, including 44 upregulated genes and 140 downregulated genes

	Ccser1	Gm3424 8	B3galt1	Slc24a3	Nxpe4	Trappc9	Tenm3	Slc39a11	Cplx2	Eda
Up-genes	Bbs9	Dennd1a	AI197445	Fhod3	Scaper	Ptprg	Ust	Dis3l2	Col4a6	Ankrd44
	Fggy	Poln	Lrba	Gtdc1	Ralgps1	Jazf1	Prkce	Tln2	Vav3	Eml6
	Enox2	Atrnl1	Gm37240	Zfp704	Msra	Macrod1	Fbxl17	3010001F23Rik	Suco	2210408F21Rik
	Kcnq5	Exoc4	Fam172a	Large1						
	Rab15	Sprr1a	B3gnt8	AC161757.1	Sptbn2	Lce1g	Sele	Lrrc15	Aldh1a3	Krt16
	B930092H01Rik	Csf2	Zfp750	H2ac6	H3c14	Esrp2	Ang2	Cbs	Col2a1	Il1rl1
	Mmp24	Serpinb2	Ctsf	Gm32200	Zscan10	Dchs1	Cntnap4	Wincr1	U90926	Crhr2
	Dusp2	H4c12	Il33	H1f3	Ptges	Mctp2	Gm50397	Gm42743	Odaph	Dcn
	Egfros	Dusp9	H2bc4	Islr2	H2aw	Sp7	Rnf183	Gm49774	Cdcp1	H1f4
	H4c14	Rcsd1	Urah	Gm10827	P2ry6	Atf3	4833427G06Rik	Mesp2	Angptl4	Fam180a
	1700017M07Rik	S1pr3	Evpl	H2bc8	Spink10	9530062K07 Rik	Mslnl	Liph	Gm9917	Hpx
	Rgs16	Has2	Tcim	Glrp1	Dpysl5	Fos	Hes2	S100a7a	Myom1	Gm42809
	H4c11	Gm26644	Wfikkn2	Acsbg1	A530013C23Rik	Rgcc	Slc5a5	Cacng1	Col6a3	Pinlyp
	H2bc3	Chrm1	H2bc9	Noxred1	Vmn1r43	Gm19801	H4c9	Ngef	Pde9a	Rab44
	Slc2a5	Tmem95	Gldc	Snx20	Hhipl1	Cnga3	Il24	E230029C05Rik	Gm20544	Nr1h3
Down-genes	Adgrb1	Tnfrsf8	Bcl2l15	Mab21l1	Prss22	Ccdc184	Gm44652	Nppb	Foxn1	Ces1a
	Gm13283	Omp	Papolb	H1f1	Gm16196	Akap5	Gm37696	Sh3bgrl2	Gm42752	Ptgs2
	Krt15	H1f2	Gm648	Krt14	Zfp972	Pm20d1	Crhbp	Rasl11a	Serpinb1b	Il11

### MCODE/Clue-GO plug-in analysis

**Fig. 6:**
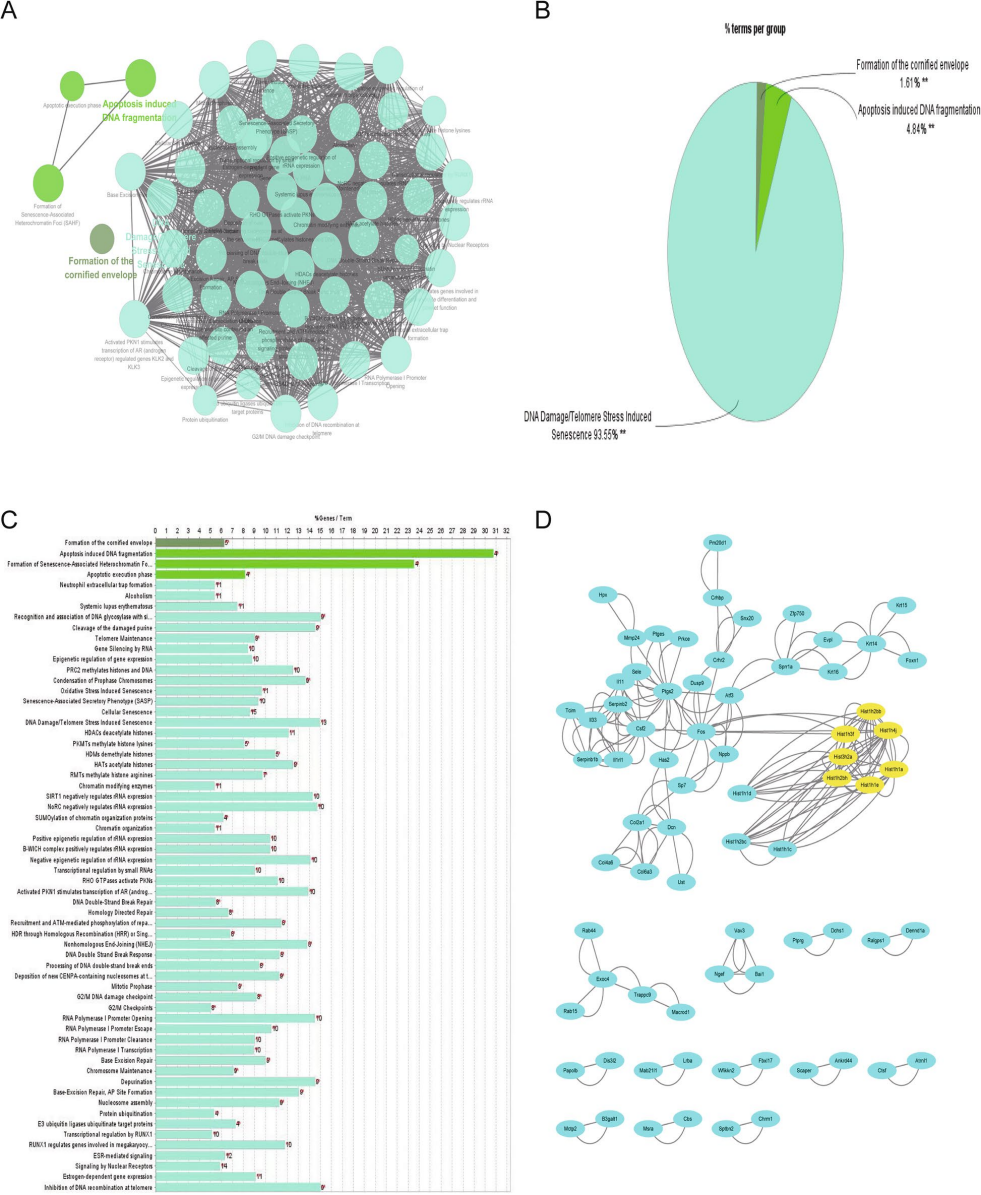
Functional enrichment analysis and pathway enrichment analysis based on cytoscape. **A** GO analysis and KEGG analysis of 184 genes based on clue-go plug-in, *P* < 0.05. **B** Pie chart showing enrichment analysis results. **C** Histogram showing enrichment analysis results. **D** Based on cytoscape software, the protein–protein interaction network output from the STRING database was further analyzed. The yellow dots represent hub genes

Using the clue-go plugin to analyze the genes, the result is that 184 genes are enriched in DNA-damage/Telomere stress induced senescence (93.55%), Apoptosis induced DNA fragmentation (4.84%), and formation of the cornified envelope (1.61%). And the analysis results are shown in the Fig. 6A-C. Using the MCODE plugin in the cytoscae software, set the threshold to degree cutoff: 12, K-Core = 4. The overall PPI network consists of 73 nodes and 204 edges. Seven hub genes (*Hist1h1e, Hist1h2bh, Hist1h4j, Hist3h2a, Hist1h1a, Hist1h3f,* and *Hist1h2bb*) were identified in key modules, and the yellow circles represent the core genes. Based on MCODE, salient modules (7 nodes 36 edges) were selected from the PPI network. The results are shown in Fig. 6D.

### RG108 downgrade cisplatin initiated ROS in HEI-OC1 cells

**Fig. 7:**
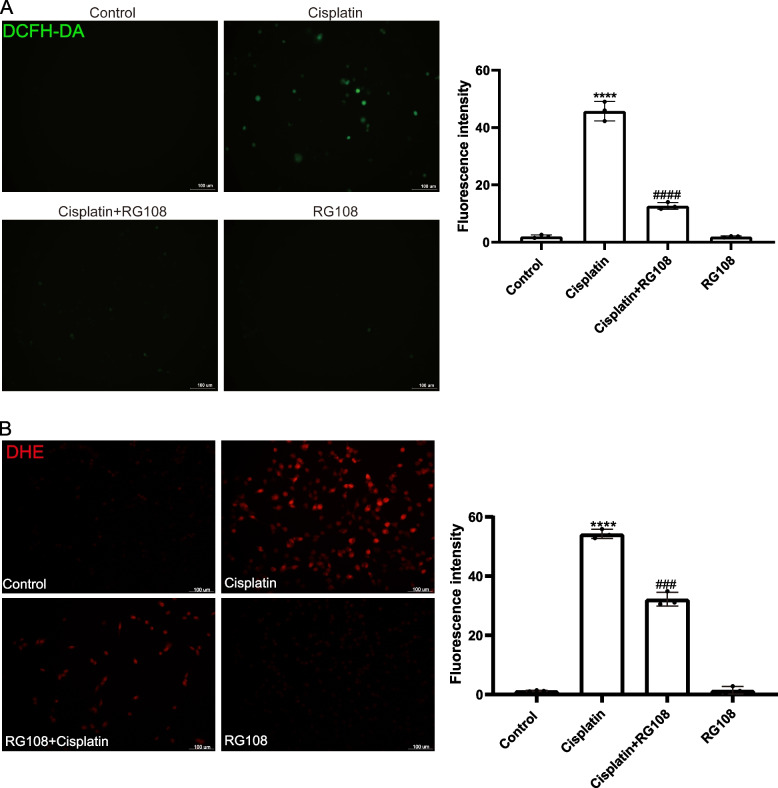
ROS detected by DCFH-DA and DHE staining. **A** Fluorescence images from four groups (DMSO, CIS, CIS + RG108, RG108) and Measure the fluorescence intensity with ImageJ software by DCFH-DA staining. Data are expressed as mean ± SD, *n* = 3,**** *P* < 0.0001 compared with the control group. Compared with cisplatin group, ^#####^*p* < 0.0001. scale: 100 μ m. **B** Fluorescence images from four groups (DMSO, CIS, CIS + RG108, RG108) and Measure the fluorescence intensity with ImageJ software by DHE staining. Data are expressed as mean ± SD, *n* = 3,**** *P* < 0.0001 compared with the control group. Compared with cisplatin group, ^####^*p* < 0.001. scale: 100 μ m

We inspected the fruitage of ROS with the mitochondrial specific ROS index DCFH-DA and DHE to inspect whether RG108 can alleviate cellular oxidative stress. It can be seen that the fruitage of ROS initiated by cisplatin is distinguished up-regulated, while the pretreatment of RG108 2 h in advance can significantly reduce the induction of ROS. Moreover, the use of RG108alone will not have an impact on ROS of HEI-OC1. Various signs show the protective effect of RG108 on cisplatin injury (Fig. 7A-B).

### RG108 alleviates cisplatin induced cochlear cell apoptosis in vitro through up-regulated PI3K / AKT pathway and down-regulated caspase3

**Fig. 8:**
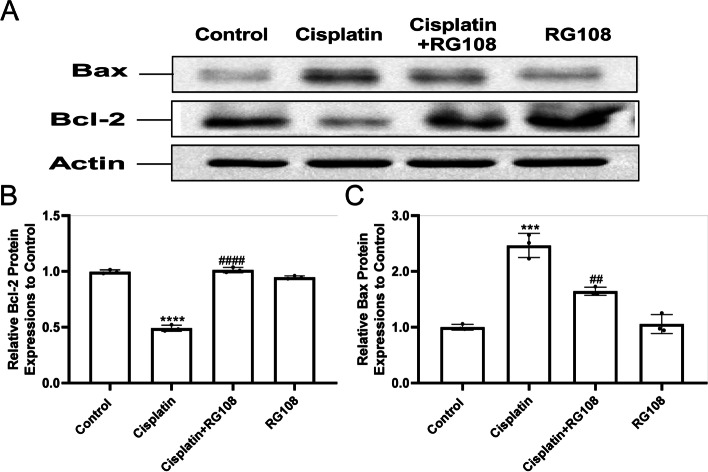
In auditory cells (HEI-OC1) cells, BAX was up-regulated and BCL2 was down-regulated after cisplatin injury, but RG108 reversed this phenomenon. **A** Western blotting was used to detect BCL2, BAX protein levels in HEI-OC1 cells treated with DMSO, CIS, CIS + RG108, RG108. **B** Use ImageJ to analyze the relative expression of BAX protein. **C** Use ImageJ to analyze the relative expression of BCL2 protein. Data are expressed as mean ± SD, *n* = 3,*** *P* < 0.001, * * * * *P* < 0.0001, compared with the control group; Compared with cisplatin group, ^##^ *P* < 0.01, ^####^ *P* < 0.0001

**Fig. 9:**
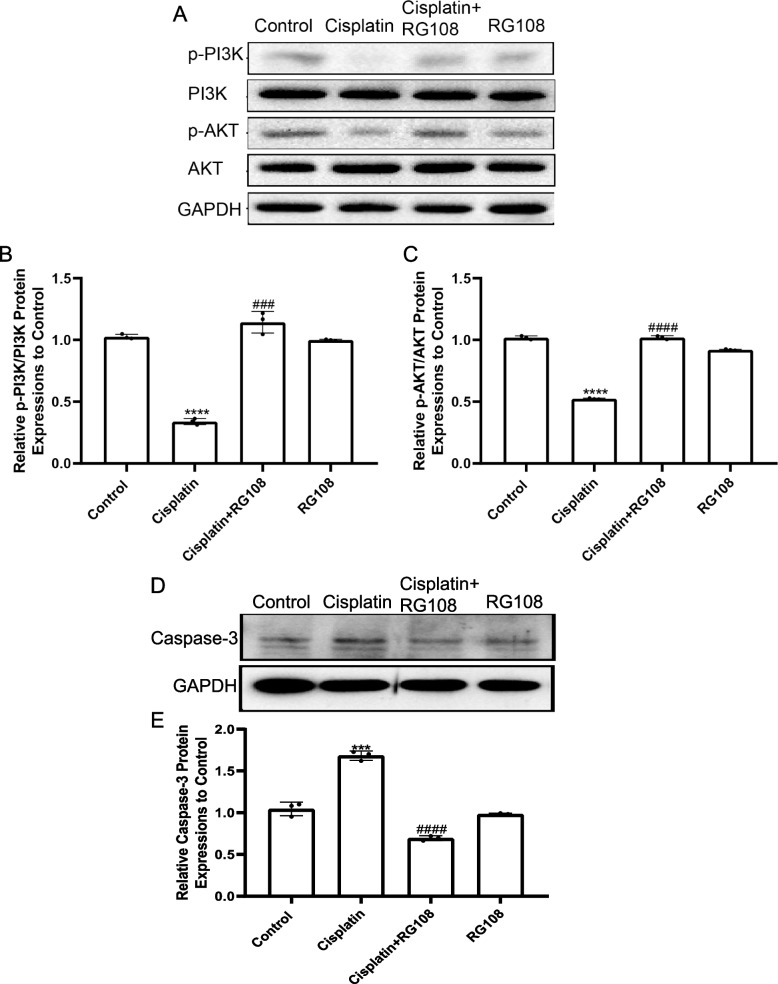
In auditory cells (HEI-OC1) cells, P-PI3K was down-regulated and P-AKT was down-regulated after cisplatin injury, but RG108 reversed this phenomenon. However, RG108 had no effect on the expression of PI3K and AKT. Caspase-3 was up-regulated after cisplatin injury, but RG108 reversed this phenomenon. **A** Western blotting was used to detect P-PI3K, P-AKT, PI3K and AKT protein levels in HEI-OC1 cells treated with DMSO, CIS, CIS + RG108, RG108. **B** Use ImageJ to analyze the relative expression of P-PI3K/PI3K protein. **C** Use ImageJ to analyze the relative expression of P-AKT/AKT protein. **D** Western blotting was used to detect Caspase-3 protein levels in HEI-OC1 cells treated with DMSO, CIS, CIS + RG108, RG108. **E** Use ImageJ to analyze the relative expression of Caspase-3 protein. Data are expressed as mean ± SD, *n* = 3,**** *P* < 0.0001, compared with the control group; Compared with cisplatin group, ^###^ *P* < 0.001, ^####^ *P* < 0.0001

**Fig. 10:**
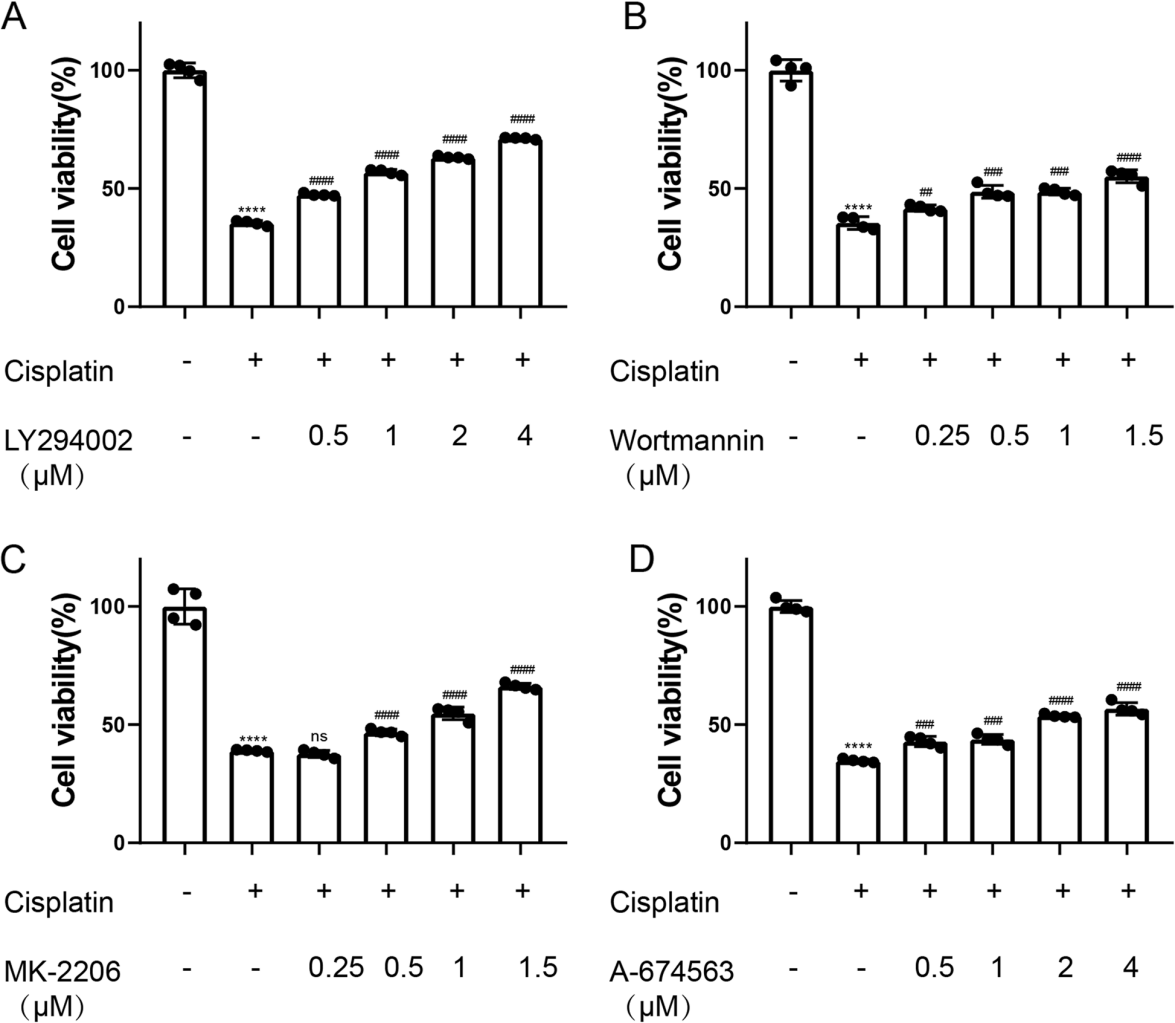
MTT assay was used to detect the difference in the activity of PI3K and AKT inhibitors co treated with cisplatin. **A** Determination of cell viability in LY294002 treatment group. **B** Determination of cell viability in Wortmannin treatment group. **C** Determination of cell viability in MK-2206 treatment group. **D** Determination of cell viability in A-674563 treatment group. Data are expressed as mean ± SD, *n* = 3,**** *P* < 0.0001 compared with the control group. Compared with cisplatin group, ^##^*P* < 0.01. ^###^ *P* < 0.001.^####^*p* < 0.0001. scale: 100 μ m

To explore the secret of RG108 on cochlear hair cell apoptosis, the expression of Bcl-2 family proteins was inspected by Western blot in cisplatin injured cells. Cisplatin induced a significant increase in Bax and a significant decrease in Bcl-2 (Fig. 8A-C). Then, we detected the main molecules of PI3K / AKT signaling pathway. Downregulation of P-PI3K and P-AKT in cisplatin injured group was detected. Encouragingly, pretreatment with RG108 can significantly up regulate P-PI3K and P-AKT. Above research confirm our results that RG108 has a strong ability to resist cisplatin induced apoptosis of HEI-OC1 cells (Fig. 9A-C). We detected the activation of Caspase-3 in the apoptotic signaling pathway, and the results were obvious. Cisplatin caused obvious activation of Caspase-3, and RG108 pretreatment could alleviate this situation (Fig. 9D).We treated the cells with PI3K and AKT inhibitors together with cisplatin. The results showed that both the PI3K inhibitors LY294002 and Wortmannin, or the AKT inhibitors MK-2206 and A-674563, could significantly rescue the damage of HEI-OC1 caused by cisplatin. It indicates that cisplatin may damage cochlear hair cells through PI3K and AKT signaling pathway (Fig. 10A-D).

### RG108 stimulates oxidative stress pathway to resist cisplatin induced HEI-OC1 injury

**Fig. 11:**
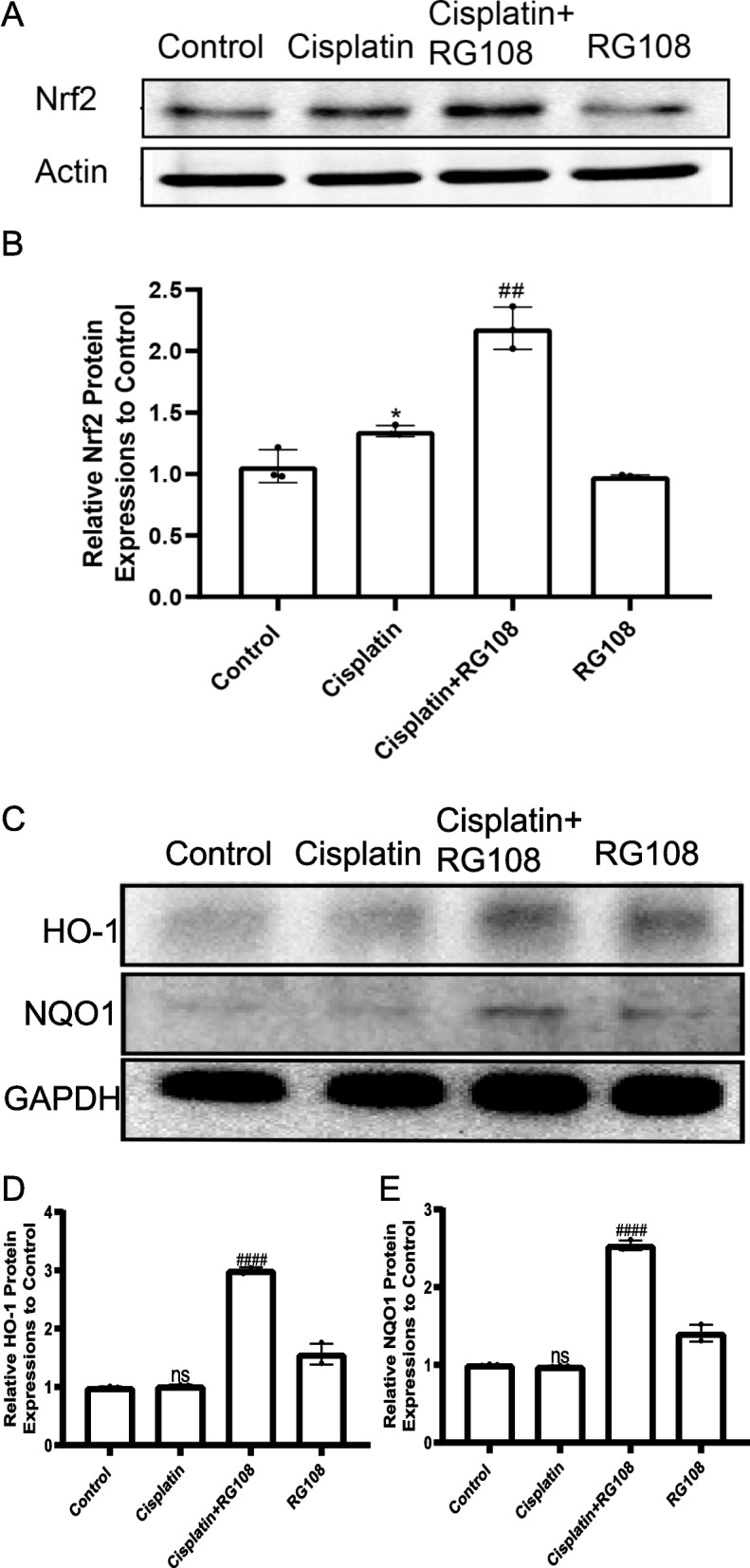
In auditory cells (HEI-OC1) cells, NRF2, HO-1 and NQO1 were up-regulated after cisplatin injury, but NRF2, HO-1 and NQO1 was obviously up-regulated after RG108 + CIS treatment. **A** Western blotting was used to detect NRF2 protein levels in HEI-OC1 cells treated with DMSO, CIS, CIS + RG108, RG108. **A** Use ImageJ to analyze the relative expression of NRF2 protein. Data are expressed as mean ± SD, *n* = 3,* *P* < 0.05 compared with the control group;^##^*P* < 0.01, compared with cisplatin group. **B** Western blotting was used to detect HO-1 and NQO1 protein levels in HEI-OC1 cells treated with DMSO, CIS, CIS + RG108, RG108. **C** Use ImageJ to analyze the relative expression of HO-1 protein. Data are expressed as mean ± SD, *n* = 3,* *P* < 0.05 compared with the control group;^##^*P* < 0.01, compared with cisplatin group. **D** Use ImageJ to analyze the relative expression of NQO1 protein. Data are expressed as mean ± SD, *n* = 3,* *P* < 0.05 compared with the control group;^##^*P* < 0.01, compared with cisplatin group

**Fig. 12:**
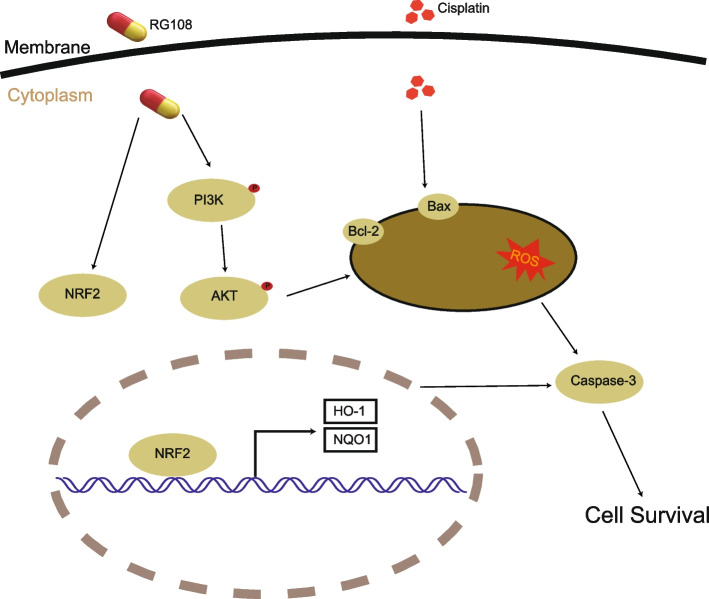
Mechanism diagram of this study

NRF2 is an antioxidant enzyme that cannot be ignored in the process of biological oxidative stress. We detected its expression. As shown in the figure, NRF2 in the cisplatin injury group increased slightly compared with the control group. We speculate that it may activate oxidative stress to resist cisplatin injury. However, importantly, NRF2 level increased significantly after RG108 pretreatment, The quantitative results of Fig. 11B also show that the difference between RG108 pretreatment and cisplatin alone injury group is significant (*P* < 0.01). These results highly suggest that the resistance of RG108 to cisplatin injury is caused by activating the expression of antioxidant enzyme NRF2 in oxidative stress (Fig. 11A-B). As for the targets of NRF2 signaling pathway, we selected HO-1 and NQO1 to detect their protein expression. It can be seen from the figure that RG108 pretreatment can significantly increase the expression of HO-1 and NQO1, which may be very important to rescue the damage of cochlear hair cells caused by Cisplatin (Fig. 11C). The mechanism diagram is shown in Fig. 12.

## Discussion

As a worldwide public health event, deafness deserves attention. By applying bioinformatics methods to analyze RG108-treated cells, we found that RG108, as a small molecule inhibitor, plays an anti-apoptotic role in drug-induced deafness and protects cochlear cells from damage, and further confirmed our Analysis results. Our results explored that RG108 plays a saviour character in DIHL.

In this study, through RNA-seq sequencing, cisplatin promotes the apoptosis of auditory cells, which is a major cause of deafness. After adding RG108. It was found that RG108 could salvation the apoptosis of HEI-OC1 induced by cisplatin. The above results showed that RG108 may play a role in apoptosis. Therefore, we continue to explore the biological mechanism that may have this effect.

We first performed biological analysis on the differentially expressed genes obtained by sequencing. This analysis used the DAVID database, the selected genes meet *P* < 0.05, | LOG fold change |≥ 2, fold change was twofold. The results showed that the down-regulated differential genes were enriched in the PI3K and NRF2 related pathways after RG108 treatment. KEGG analysis showed that down-regulated differential genes were enriched in PI3K, which was an apoptosis-related pathway. BIOCARTA analysis showed that down-regulated differential genes were enriched in NRF2 oxidative stress-related pathway, which was closely related to apoptosis. We further analyzed the DEGs using the STRING database, the selected genes met *P* < 0.05, | LOG fold change |≥ 3, fold change was eightfold. The results were further analyzed using the Clue-GO plug-in, and finally the DEGs were enriched in apoptosis induced DNA fragmentation. DEGs are associated with apoptosis-related pathways, and it can be further indicate that RG108 has an effect on cisplatin-induced apoptosis pathways.

Through a comprehensive analysis of the above DAVID, KEGG, MCODE / Clue-GO, STRING, BIOCARTA databases, we determined that rg108 affected the cisplatin-induced apoptosis pathway.ROS not only participates in apoptosis and necrosis, but also participates in intercellular signal transduction and affects gene expression [50]. The accumulation of ROS and the subsequent induction of apoptosis are important factors leading to various diseases and aging [51]. The content of ROS leads to changes in the biological state of cochlear cells, which in turn leads to the loss of hair cells. Xiangrui Guo's research team showed that Forskolin protects cochlear hair cells from apoptosis and acts as an antioxidant [52]. Our findings suggest that RG108 also exerts an anti-apoptotic effect in cochlear cells. Therefore, we predict that RG108 can be used as a clinical trial drug for deaf patients. BAX, BCL2 is an apoptosis-related protein. When the expression level changes, it indicates that the cells have the phenomenon of promoting or inhibiting apoptosis. Tengfei Zhao’s team study showed that ligustrazine extracted from Ligusticum chuanxiong Hort had an effect on apoptosis by changing the expression of BAX and BCL2. BCL2 inhibits apoptosis, and BAX promotes apoptosis [53]. Our results showed that RG108 upregulated BCL2 and downregulated BAX. Therefore, we speculated that RG108 might have a protective effect on apoptosis by affecting BAX and BCL2. By examining the level of important proteins in the PASP, and cisplatin could noticeably reduce the expression of phosphorylated PI3K-AKT, it had no effect on the expression of PI3K-ATK as a whole. This suggests that RG108 may interfere with cochlear apoptosis by regulating the expression of phosphorylated protein. RG108 treatment also increased the expression of phosphorylated PI3K-AKT, but still had no effect on the level of total PI3K-ATK. RG108 treatment also increased the expression of NRF2. Numerous studies have reported that NRF2 is involved in oxidative stress [54–56]. Caspase-3 is the key substance of apoptosis and plays an important role in ROS. This study found that RG108 can down-regulate caspase3, protect cochlear cell apoptosis and protect the occurrence of drug-induced deafness [57]. We observed that RG108 could upregulate NRF2 protein levels in cisplatin-induced cells. It is well known that NRF2 can reduce oxidative stress-induced cell damage and maintain the homeostasis of the redox system by derivating and modulating the level of various antioxidant factors. HO-1 and NQO1 are important factors in the NRF2 pathway, and the expression of these two proteins decreases when cells undergo apoptosis. We observed that RG108 treatment significantly up-regulated the protein expressions of HO-1 and NQO1, indicating that RG108 exerted a protective effect on cisplatin-induced apoptosis by affecting HO-1 and NQO1 in the NRF2 pathway [58].

Therefore, we believe that RG108 can alleviate cisplatin-induced oxidative stress damage of cells, especially in the case of pretreatment, which is also obvious for clinical implications, that is, the intervention of RG108 in advance may reduce cisplatin-induced deafness. However, this study still has many shortcomings. Next, we will try to find the downstream molecular target of RG108, and further explore the molecular mechanism of RG108 to save the apoptosis of cochlear hair cells induced by cisplatin, and whether it interferes with the clinical efficacy of cisplatin, so as to provide ideas for clinical trials.

## Conclusion

In summary, this study found that RG108 may have a preserve effect on HEI-OC1 damaged by cisplatin by affecting NRF2 / PI3K-AKT axis. As a result of this study, RG108 can now be applied to the treatment of cisplatin ototoxicity. At the same time, it also creates a research direction for subsequent clinical trials.

## Data Availability

All raw data for this study are available from the corresponding authors. The BioProject number: PRJNA872323. Sequence Read Archive (SRA) submission: SUB11970562.

## Abbreviation

DIHL: Drug-induced hearing loss
GO: Gene ontology
PASP: PI3K-AKT signaling pathway
WB: Western blotting
HL: Hearing loss
CIS: Cisplatin
DCFH-DA: 2',7'-Dichlorodihydrofluorescein diacetate
DHE: Dihydroethidium
DEGs: Differentially expressed genes
NGF: Nerve growth factor pathway

## References

[1] Yasui N, Adachi N, Kato M, Koh K, Asanuma S, Sakata H, Hanada R (2014). Cisplatin-induced hearing loss: the need for a long-term evaluating system. J Pediatr Hematol Oncol.

[2] Nieman CL, Oh ES (2020). Hearing Loss. Ann Intern Med.

[3] Ghosh S (2019). Cisplatin: The first metal based anticancer drug. Bioorg Chem.

[4] Dasari S, Tchounwou PB (2014). Cisplatin in cancer therapy: molecular mechanisms of action. Eur J Pharmacol.

[5] Wang H, Guo S, Kim SJ, Shao F, Ho JWK, Wong KU, Miao Z, Hao D, Zhao M, Xu J, Zeng J, Wong KH, Di L, Wong AH, Xu X, Deng CX (2021). Cisplatin prevents breast cancer metastasis through blocking early EMT and retards cancer growth together with paclitaxel. Theranostics.

[6] Sousa DP, Pojo M, Pinto AT, Leite V, Serra AT, Cavaco BM (2020). Nobiletin Alone or in Combination with Cisplatin Decreases the Viability of Anaplastic Thyroid Cancer Cell Lines. Nutr Cancer.

[7] Federico C, Sun J, Muz B, Alhallak K, Cosper PF, Muhammad N, Jeske A, Hinger A, Markovina S, Grigsby P, Schwarz JK, Azab AK (2021). Localized Delivery of Cisplatin to Cervical Cancer Improves Its Therapeutic Efficacy and Minimizes Its Side Effect Profile. Int J Radiat Oncol Biol Phys.

[8] Huang Y, Lei L, Liu Y (2020). Propofol Improves Sensitivity of Lung Cancer Cells to Cisplatin and Its Mechanism. Med Sci Monit.

[9] Jian B, Pang J, Xiong H, Zhang W, Zhan T, Su Z, Lin H, Zhang H, He W, Zheng Y (2021). Autophagy-dependent ferroptosis contributes to cisplatin-induced hearing loss. Toxicol Lett.

[10] Tang Q, Wang X, Jin H, Mi Y, Liu L, Dong M, Chen Y, Zou Z (2021). Cisplatin-induced ototoxicity: Updates on molecular mechanisms and otoprotective strategies. Eur J Pharm Biopharm.

[11] Disease GBD, Injury I, Prevalence C (2016). Global, regional, and national incidence, prevalence, and years lived with disability for 310 diseases and injuries, 1990–2015: a systematic analysis for the Global Burden of Disease Study 2015. Lancet.

[12] Hoffmann TJ, Keats BJ, Yoshikawa N, Schaefer C, Risch N, Lustig LR (2016). A Large Genome-Wide Association Study of Age-Related Hearing Impairment Using Electronic Health Records. PLoS Genet.

[13] Golub JS, Luchsinger JA, Manly JJ, Stern Y, Mayeux R, Schupf N (2017). Observed Hearing Loss and Incident Dementia in a Multiethnic Cohort. J Am Geriatr Soc.

[14] Lanvers-Kaminsky C, Zehnhoff-Dinnesen AA, Parfitt R, Ciarimboli G (2017). Drug-induced ototoxicity: Mechanisms, Pharmacogenetics, and protective strategies. Clin Pharmacol Ther.

[15] Ruan M, Cheng Q, Gong C, Cao Z, Xu L, Zhang Q (2020). Development of a kind of RG108-Fluorescein conjugates for detection of DNA methyltransferase 1 (DNMT1) in living cells. Anal Biochem.

[16] Zheng Z, Zeng S, Liu C, Li W, Zhao L, Cai C, Nie G, He Y (2021). The DNA methylation inhibitor RG108 protects against noise-induced hearing loss. Cell Biol Toxicol.

[17] Elmore S (2007). Apoptosis: a review of programmed cell death. Toxicol Pathol.

[18] Ruhl D, Du TT, Wagner EL, Choi JH, Li S, Reed R, Kim K, Freeman M, Hashisaki G, Lukens JR, Shin JB (2019). Necroptosis and Apoptosis Contribute to Cisplatin and Aminoglycoside Ototoxicity. J Neurosci.

[19] Ma Q (2013). Role of nrf2 in oxidative stress and toxicity. Annu Rev Pharmacol Toxicol.

[20] Cuadrado A, Manda G, Hassan A, Alcaraz MJ, Barbas C, Daiber A, Ghezzi P, Leon R, Lopez MG, Oliva B, Pajares M, Rojo AI, Robledinos-Anton N, Valverde AM, Guney E, Schmidt H (2018). Transcription Factor NRF2 as a Therapeutic Target for Chronic Diseases: A Systems Medicine Approach. Pharmacol Rev.

[21] Sykiotis GP, Bohmann D (2010). Stress-activated cap'n'collar transcription factors in aging and human disease. Sci Signal.

[22] Jaramillo MC, Zhang DD (2013). The emerging role of the Nrf2-Keap1 signaling pathway in cancer. Genes Dev.

[23] Li W, Khor TO, Xu C, Shen G, Jeong WS, Yu S, Kong AN (2008). Activation of Nrf2-antioxidant signaling attenuates NFkappaB-inflammatory response and elicits apoptosis. Biochem Pharmacol.

[24] Fetoni AR, Paciello F, Mezzogori D, Rolesi R, Eramo SL, Paludetti G, Troiani D (2015). Molecular targets for anticancer redox chemotherapy and cisplatin-induced ototoxicity: the role of curcumin on pSTAT3 and Nrf-2 signalling. Br J Cancer.

[25] Kim SJ, Ho Hur J, Park C, Kim HJ, Oh GS, Lee JN, Yoo SJ, Choe SK, So HS, Lim DJ, Moon SK, Park R (2015). Bucillamine prevents cisplatin-induced ototoxicity through induction of glutathione and antioxidant genes. Exp Mol Med.

[26] Honkura Y, Matsuo H, Murakami S, Sakiyama M, Mizutari K, Shiotani A, Yamamoto M, Morita I, Shinomiya N, Kawase T, Katori Y, Motohashi H (2016). NRF2 Is a Key Target for Prevention of Noise-Induced Hearing Loss by Reducing Oxidative Damage of Cochlea. Sci Rep.

[27] Xie Y, Shi X, Sheng K, Han G, Li W, Zhao Q, Jiang B, Feng J, Li J, Gu Y (2019). PI3K/Akt signaling transduction pathway, erythropoiesis and glycolysis in hypoxia (Review). Mol Med Rep.

[28] Martini M, De Santis MC, Braccini L, Gulluni F, Hirsch E (2014). PI3K/AKT signaling pathway and cancer: an updated review. Ann Med.

[29] Xu C, Huang X, Huang Y, Liu X, Wu M, Wang J, Duan X (2021). Naringin induces apoptosis of gastric carcinoma cells via blocking the PI3K/AKT pathway and activating prodeath autophagy. Mol Med Rep.

[30] Yan HZ, Wang HF, Yin Y, Zou J, Xiao F, Yi LN, He Y, He BS (2021). GHR is involved in gastric cell growth and apoptosis via PI3K/AKT signalling. J Cell Mol Med.

[31] Nozaki T, Kanai M (2021). Chemical Catalysis Intervening to Histone Epigenetics. Acc Chem Res.

[32] Pareek CS, Smoczynski R, Tretyn A (2011). Sequencing technologies and genome sequencing. J Appl Genet.

[33] Behjati S, Tarpey PS (2013). What is next generation sequencing?. Arch Dis Child Educ Pract Ed.

[34] Kalinec GM, Webster P, Lim DJ, Kalinec F (2003). A cochlear cell line as an in vitro system for drug ototoxicity screening. Audiol Neurootol.

[35] Ou Y, Zhang Q, Tang Y, Lu Z, Lu X, Zhou X, Liu C (2018). DNA methylation enzyme inhibitor RG108 suppresses the radioresistance of esophageal cancer. Oncol Rep.

[36] Yang L, Hou J, Cui XH, Suo LN, Lv YW (2017). RG108 induces the apoptosis of endometrial cancer Ishikawa cell lines by inhibiting the expression of DNMT3B and demethylation of HMLH1. Eur Rev Med Pharmacol Sci.

[37] Graca I, Sousa EJ, Baptista T, Almeida M, Ramalho-Carvalho J, Palmeira C, Henrique R, Jeronimo C (2014). Anti-tumoral effect of the non-nucleoside DNMT inhibitor RG108 in human prostate cancer cells. Curr Pharm Des.

[38] Gerda de Vries G, Rosas-Plaza X, van Vugt M, Gietema JA, de Jong S (2020). Testicular cancer: Determinants of cisplatin sensitivity and novel therapeutic opportunities. Cancer Treat Rev.

[39] Deng Y, Guo W, Xu N, Li F, Li J (2020). CtBP1 transactivates RAD51 and confers cisplatin resistance to breast cancer cells. Mol Carcinog.

[40] Kiss RC, Xia F, Acklin S (2021). Targeting DNA Damage Response and Repair to Enhance Therapeutic Index in Cisplatin-Based Cancer Treatment. Int J Mol Sci.

[41] Mitsudomi T, Morita S, Yatabe Y, Negoro S, Okamoto I, Tsurutani J, Seto T, Satouchi M, Tada H, Hirashima T, Asami K, Katakami N, Takada M, Yoshioka H, Shibata K, Kudoh S, Shimizu E, Saito H, Toyooka S, Nakagawa K, Fukuoka M, Oncology WJ, G.  (2010). Gefitinib versus cisplatin plus docetaxel in patients with non-small-cell lung cancer harbouring mutations of the epidermal growth factor receptor (WJTOG3405): an open label, randomised phase 3 trial. Lancet Oncol.

[42] Layeghifard M, Hwang DM, Guttman DS (2018). Constructing and Analyzing Microbiome Networks in R. Methods Mol Biol.

[43] da Huang W, Sherman BT, Lempicki RA (2009). Systematic and integrative analysis of large gene lists using DAVID bioinformatics resources. Nat Protoc.

[44] Ashburner M, Ball CA, Blake JA, Botstein D, Butler H, Cherry JM, Davis AP, Dolinski K, Dwight SS, Eppig JT, Harris MA, Hill DP, Issel-Tarver L, Kasarskis A, Lewis S, Matese JC, Richardson JE, Ringwald M, Rubin GM, Sherlock G (2000). Gene ontology: tool for the unification of biology. The Gene Ontology Consortium. Nat Genet.

[45] Gene Ontology C (2006). The Gene Ontology (GO) project in 2006. Nucleic Acids Res.

[46] Kanehisa M, Goto S (2000). KEGG: kyoto encyclopedia of genes and genomes. Nucleic Acids Res.

[47] Szklarczyk D, Gable AL, Nastou KC, Lyon D, Kirsch R, Pyysalo S, Doncheva NT, Legeay M, Fang T, Bork P, Jensen LJ, von Mering C (2021). The STRING database in 2021: customizable protein-protein networks, and functional characterization of user-uploaded gene/measurement sets. Nucleic Acids Res.

[48] Otasek D, Morris JH, Boucas J, Pico AR, Demchak B (2019). Cytoscape Automation: empowering workflow-based network analysis. Genome Biol.

[49] Sepulveda JL (2020). Using R and Bioconductor in Clinical Genomics and Transcriptomics. J Mol Diagn.

[50] Luo Z, Xu X, Sho T, Zhang J, Xu W, Yao J, Xu J (2019). ROS-induced autophagy regulates porcine trophectoderm cell apoptosis, proliferation, and differentiation. Am J Physiol Cell Physiol.

[51] Orr WC, Sohal RS (1994). Extension of life-span by overexpression of superoxide dismutase and catalase in Drosophila melanogaster. Science.

[52] Guo X, Bai X, Li L, Li J, Wang H (2018). Forskolin protects against cisplatin-induced ototoxicity by inhibiting apoptosis and ROS production. Biomed Pharmacother.

[53] Zhao T, Fu Y, Sun H, Liu X (2018). Ligustrazine suppresses neuron apoptosis via the Bax/Bcl-2 and caspase-3 pathway in PC12 cells and in rats with vascular dementia. IUBMB Life.

[54] Li D, Zhao H, Cui ZK, Tian G (2021). The Role of Nrf2 in Hearing Loss. Front Pharmacol.

[55] Gentilin E, Simoni E, Candito M, Cazzador D, Astolfi L (2019). Cisplatin-Induced Ototoxicity: Updates on Molecular Targets. Trends Mol Med.

[56] Hoshino T, Tabuchi K, Nishimura B, Tanaka S, Nakayama M, Ishii T, Warabi E, Yanagawa T, Shimizu R, Yamamoto M, Hara A (2011). Protective role of Nrf2 in age-related hearing loss and gentamicin ototoxicity. Biochem Biophys Res Commun.

[57] Porter AG, Jänicke RU (1999). Emerging roles of caspase-3 in apoptosis. Cell Death Differ.

[58] Yang F, Ruixia C, Zeyu L, Xia Z, Yifan J, Xing Z, Jinghong S, Kai Q, Chang L, Jingyao Z (2019). Methane Alleviates Acetaminophen-Induced Liver Injury by Inhibiting Inflammation, Oxidative Stress, Endoplasmic Reticulum Stress, and Apoptosis through the Nrf2/HO-1/NQO1 Signaling Pathway. Oxid Med Cell Longev.

